# What are the risks of manual treatment of the spine? A scoping review for clinicians

**DOI:** 10.1186/s12998-017-0168-5

**Published:** 2017-12-07

**Authors:** Gabrielle Swait, Rob Finch

**Affiliations:** The Royal College of Chiropractors, Chiltern Chambers, St. Peters Avenue, Reading, RG4 7DH UK

**Keywords:** Adverse events, Risks, Manipulation, Chiropractic, Osteopathy, Manual therapy, Spine, Cervical, Vertebral artery, Incident reporting

## Abstract

**Background:**

Communicating to patients the risks of manual treatment to the spine is an important, but challenging element of informed consent. This scoping review aimed to characterise and summarise the available literature on risks and to describe implications for clinical practice and research.

**Method:**

A methodological framework for scoping reviews was followed. Systematic searches were conducted during June 2017. The quantity, nature and sources of literature were described. Findings of included studies were narratively summarised, highlighting key clinical points.

**Results:**

Two hundred and fifty articles were included. Cases of serious adverse events were reported. Observational studies, randomised studies and systematic reviews were also identified, reporting both benign and serious adverse events.

Benign adverse events were reported to occur commonly in adults and children. Predictive factors for risk are unclear, but for neck pain patients might include higher levels of neck disability or cervical manipulation. In neck pain patients benign adverse events may result in poorer short term, but not long term outcomes.

Serious adverse event incidence estimates ranged from 1 per 2 million manipulations to 13 per 10,000 patients. Cases are reported in adults and children, including spinal or neurological problems as well as cervical arterial strokes. Case-control studies indicate some association, in the under 45 years age group, between manual interventions and cervical arterial stroke, however it is unclear whether this is causal. Elderly patients have no greater risk of traumatic injury compared with visiting a medical practitioner for neuro-musculoskeletal problems, however some underlying conditions may increase risk.

**Conclusion:**

Existing literature indicates that benign adverse events following manual treatments to the spine are common, while serious adverse events are rare. The incidence and causal relationships with serious adverse events are challenging to establish, with gaps in the literature and inherent methodological limitations of studies. Clinicians should ensure that patients are informed of risks during the consent process. Since serious adverse events could result from pre-existing pathologies, assessment for signs or symptoms of these is important. Clinicians may also contribute to furthering understanding by utilising patient safety incident reporting and learning systems where adverse events have occurred.

**Electronic supplementary material:**

The online version of this article (10.1186/s12998-017-0168-5) contains supplementary material, which is available to authorized users.

## Background

Great emphasis is placed on the importance of patient-informed choice. Health policy in the United Kingdom states that patients have a right to be given a clear explanation of any treatment proposed, including any risks and alternatives, before they decide whether to agree to treatment [1]. There is evidence of therapeutic benefit whereby, when patients are effectively informed and can exert knowledgeable control over their treatment choices, recovery and pain tolerance may be enhanced, depression prevented, cooperation increased [2] and costs reduced [3].

The question of the risks of manual treatment of the spine, as normally provided by chiropractors, osteopaths and physiotherapists, is a much-debated issue. It has been clearly reported that the risk of major adverse events following manual therapy interventions is low [4], but some argue that the potential for serious harm following some treatment approaches poses an unacceptable risk [5, 6]. Clinicians need to meet the challenge of effectively communicating both the potential benefits and possible risks of proposed interventions. With such opposing views, it may be difficult for clinicians to understand what the existing literature does and does not tell us about risks. While systematic reviews exist for some specific questions about risks of care [4, 7–12], there has been no broad review that facilitates understanding by clinicians across the subject. The purpose of this scoping review is to map the current literature on safety and risks of manual treatment of the spine in order to identify types and sources of evidence and gaps in the research [13]. There is an emphasis on identifying points with implications for clinical practice [14].

Definitions of what constitutes a ‘risk’ of treatment vary but, in the medical literature, the term ‘adverse event’ is used to refer to iatrogenic occurrences following care. These are untoward, undesirable or detrimental, have an impact on the patient and are caused by a healthcare process rather than the natural process of disease [15]. Further categorisation of the adverse event is usually based on its severity or time course. For manual therapies, a consensus categorisation has been proposed whereby ‘major’ adverse events are medium to long-term, moderate to severe and unacceptable; they normally require further treatment and are serious and distressing. ‘Moderate’ adverse events are as major adverse events but moderate in severity. ‘Mild’ events are short-term, non-serious, the patient’s function remains intact, and they are transient/reversible; no treatment alterations are required because the consequences are short-term and contained [16]. These mild events are often referred to in the literature as ‘benign adverse events’. However, in the literature we reviewed, categorisation does not necessarily map to the above definitions [17]. For the purpose of this review, adverse events are therefore dichotomised into ‘benign’ (mild to moderate, transient) and ‘serious’ (moderate to major, long-term) adverse events.

## Review methodology

The review followed a methodological framework recommended for scoping reviews [18].

### Identification of the research question

The review was broad in scope and evaluated the question, ‘What are the risks of manual treatment of the spine?’. This question was identified by the Council of the Royal College of Chiropractors of the United Kingdom (https://rcc-uk.org) in response to, and informed by, requests from its clinician members.

### Identification of relevant studies

Searches were carried out by one author (GS) of MEDLINE (1946-current), EMBASE (1947-current) and of the Cochrane Library in June 2017, using search terms relating to ‘chiropractic, osteopathy, manual therapy, spinal manipulation and spinal mobilisation’ combined with search terms relating to ‘safety, risk, side-effects, adverse events, harm, death, and also to specific conditions (‘dural tear, intra-cranial hypotension, stroke, cervical artery, vertebral artery, carotid artery, paralysis, quadriplegia, Brown Sequard and cauda equina syndrome)’. An example search strategy is provided in Additional file 1: Appendix 1. The related articles search features were used and bibliographies of all relevant articles were scrutinised.

### Study selection

Retrieved articles were screened and evaluated for eligibility by one author (GS). Criteria for inclusion and exclusion of studies are provided in Table 1. Retrieved references were exported into EndNote X7 (Thomson Reuters, New York, NY, USA). Titles and abstracts were screened. Full text of potentially relevant articles was obtained and evaluated for eligibility.

**Table 1:** Eligibility criteria for inclusion and exclusion of studies

Criteria for study inclusion	Criteria for study exclusion
Participants	
• Patients receiving spinal manual treatment • Health or legal professionals reporting upon patients receiving spinal manual treatment	
Study design	Article type
• Studies whose primary aims address risks of care &/or adverse events & that are: • Meta-analyses • Systematic reviews^a^ • Controlled studies • Surveys • Cohort studies • Case reports^b^ • Scientific reports^c^	• Reviews (without a systematic approach)^d^ • Editorials, commentaries or opinion articles^d^ • Letters^e^, correspondences or author responses • Studies whose primary aims address clinical outcomes (but may report occurrence of adverse events)^f^ • Study protocols • Conference abstracts^g^
Intervention	Intervention
• Spinal manual treatment (manipulation or mobilisation), provided by a health professional	• Patient self-manipulation • Spinal manual treatment provided by a lay-person
Outcomes	Outcomes
• Adverse events	• Biomechanical or physiological responses as proxy adverse effects

^a^Reviews describing a systematically approached methodology

^b^Published as articles, letters or conference abstracts (enabling full breadth of types of adverse events to be evaluated)

^c^Describing a rigorous methodology

^d^Would contribute limited new insights to the literature reviewed

^e^Unless presenting a new case-report

^f^Limited utility for gaining new insights if not a primary consideration in study design

^g^Excluding case reports, where conference abstracts were included

### Charting data

The quantity, nature and sources of literature were described. Data from eligible studies were extracted by the first reviewer into separate fields of a Microsoft Excel (2013) spreadsheet (Microsoft, Redmond, WA 98052–6399), to enable sorting and grouping by author, date, study design, number of included patients, patient characteristics (age, gender and condition for which seeking care), discipline of treating manual therapist, intervention used, comparison intervention (if any), nature of adverse event/outcome and results reported). Additional categorisation was carried out to enable further sorting into groups of related studies. This included the spinal level treated, any special age group of patients (elderly or child) and whether the adverse event was categorised as benign or serious.

### Collating, summarising and reporting results

Data were sorted to enable synthesis and narrative summarisation of reported findings in the two key categories of benign and serious adverse events. Within these, data were further sorted and summarised, where appropriate, according to the spinal level treated, intervention specified, type of adverse event, study design and whether studies reported on elderly patients or children. Findings are presented as ‘manual intervention to the spine’ where manipulation and mobilisation may both be included. Reference to the type of manual therapist providing care is reserved for instances where ‘visits’, as opposed to a specific intervention, are reported in the literature. Summary points for clinicians to consider in their communication of risk to patients are provided below each section.

## Review findings

### What literature exists on the risks of manual treatments to the spine?

**Fig. 1:**
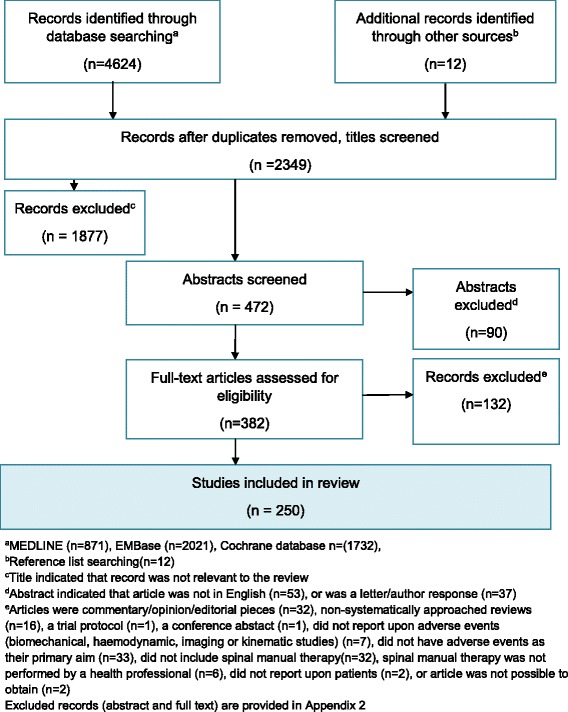
PRISMA flow chart of search results showing sources of records and exclusions at each stage of the review

Figure 1 provides the results of the literature searches and assessment for eligibility for inclusion in the review, including reasons for exclusions. Studies excluded following screening of abstracts and following evaluation of full-text articles are listed in Additional file 2: Appendix 2.

A total of 250 studies were included. The great majority of these (*n* = 166) were reports of serious adverse events in case-reports, case-series or retrospective case reviews (including several reviews of cases captured in medico-legal records [19–23]) [19–184]. These were regularly published as ‘case-reports’ in the form of letters in medical journals (*n* = 19) [185–203] or as abstracts of cases presented at conferences (*n* = 17) [25, 26, 29, 31, 32, 42, 46, 59, 66, 68, 77, 92, 107, 112, 115, 145, 148].

Thirty four articles, reporting on 31 observational studies, collected patient data prospectively or retrospectively relating to the incidence, nature or predictive factors for the occurrence of benign or serious adverse events following manual treatment to the spine [204–237]. These included 8 case-control or case-crossover analyses examining association between serious adverse events and spinal manual treatment, all investigating cervical arterial strokes [208–211, 214, 221, 226, 233]. An additional 5 studies surveyed neurologists [238] or manual therapists/manual medicine physicians [78, 239–241] about adverse events in their patients that they reported to have occurred following manual treatment to the spine.

Six experimental studies (randomised controlled trials) were reported [242–247]. These had primary aims of evaluating the occurrence of adverse events following manipulation, compared with comparator/control interventions.

A substantial quantity of secondary research (*n* = 43), in the form of systematically approached reviews [248–280], clinical practice guidelines [281–283] and a scientific report [284], was identified in the literature. Most of this research adopted a broad approach, including studies of varied methodological designs. However, 15 were reviews that only included case reports describing serious adverse events [254, 257–259, 261, 264, 266, 267, 272–274, 278]. A few systematic reviews (*n* = 7) carried out meta-analysis, pooling of data or other analytic synthesis of findings of included studies [249, 251–253, 272]. One recent systematic review of systematic reviews was identified [270].

### Summary of findings reported in the literature

#### Occurrence of benign adverse events

Benign adverse events were reported to occur frequently following manual interventions to the spine [204, 207, 215, 222–225, 227, 228, 230–232, 234, 237, 242–244, 249, 250, 253, 256, 260, 262, 263, 276, 282]. A number of randomised-controlled trials (RCTs) [242–247, 285] and non-randomised prospective studies [204, 207, 215, 222, 225, 228, 230–232, 234] report that benign adverse events occurred in 23–83% of adult patients. The lowest incidence was reported in an RCT of patients with migraine, treated using a specific chiropractic manual thrust technique (Gonstead) [242], and the highest incidence was reported in an online patient survey following treatment in an osteopathic teaching clinic [225]. The remaining studies consistently reported an incidence of benign adverse events of approximately 30–50% following manual treatment for back and/or neck pain. Sample sizes among the RCTs ranged from 70 to 767 and, for prospective cohort studies, from 68 to 19,722. No serious adverse events were observed in these studies. A systematic review that pooled data from existing RCTs and cohort studies (including studies with primarily clinical outcomes that reported adverse event rate) estimated an incidence of mild-moderate transient adverse events of approximately 22–41% following manual therapy (not limited to spinal treatment). A further recent systematic review and meta-analysis graded the quality of the body of evidence as ‘high’ that spinal manipulation is commonly associated with transient, minor, musculoskeletal harms [271].

Benign adverse events were reported to be transient, and most commonly consisted of increased musculoskeletal pain or discomfort [204, 207, 215, 222, 225, 228, 231, 232, 234, 242], stiffness [247] and headache [207, 234, 242, 247]. Other benign adverse events reported in patients who received treatment for neck pain included feeling tired [228], faint, dizzy or lightheaded [229, 234] and tingling or numbness in the upper limb [228]. The intensity of adverse events was predominantly minor or moderate [222, 231, 232, 246] although more intense or severe transient adverse events have been reported in 5–13% of patients [215, 228]. Comparable levels of benign adverse events (29%) were reported among patients receiving chiropractic care for scoliosis [237], while lower incidence (23%) was reported among migraineurs [242] and among infants under 3 years of age (1%) (however, dependence on parental reporting makes direct comparison with studies of adults problematic). The majority of transient side-effects were reported to resolve within 24 h [207, 215, 222, 224, 231, 232].

#### Box 1. The occurrence of benign adverse events following manual interventions to the spine: Summary of implications for clinical practice.

 • Benign adverse events are common, affecting 23–83% of adult patients;

 • These are mostly mild-moderate, transient (usually resolve within 24 h) and commonly include musculoskeletal pain, stiffness and headache;

 • Dizziness, tiredness, feeling faint/lightheaded or tingling in the arms might also be experienced following neck treatment.

#### Predicting benign adverse events

Few studies have evaluated factors that may enable prediction of the occurrence of benign adverse events in patients following manual interventions to the spine. However, in one neck pain study, it was reported that a moderate-severe level of disability at baseline was associated with greater likelihood (odds ratio = 3.15, 95% confidence interval 1.01–9.80) of adverse neurologic symptoms (dizziness, nausea, visual disturbance, tinnitus, extremity weakness or confusion) following chiropractic care [244].

The contribution of specific manual interventions to benign adverse events is also poorly understood, with conflicting results reported among randomised studies [244, 246, 247]. One RCT found that cervical manipulation increased the risk of any sort of adverse event, of any severity (although none were deemed serious) and commencing at any time point following treatment, compared with cervical mobilisation [244]. Effect estimates were greater when adverse events (neck pain, stiffness/soreness, radiating pain, tiredness/fatigue, headache or neurologic symptoms) rated 2 or higher on a Numeric Rating Scale, and where these symptoms commenced within 24 h following treatment. However, another study found that, for patients with spinal pain in any region, there was no increased risk for adverse event occurrence, onset or duration following manipulation compared with a sham intervention [247]. Similarly, a further RCT, evaluating different components of manual therapy, reported that the incidence of benign adverse events was no different when either manipulation or stretching were excluded from a multimodal intervention [246]. These findings raise the possibility that adverse events may, at least in part, be due to non-specific effects or to natural progression of symptoms rather than to spinal manipulation. A systematic review and meta-analysis was able to perform limited pooling of RCT data to evaluate some adverse events following cervical spinal manipulation, compared with either cervical mobilisation or manipulation elsewhere in the spine (thoracic). They reported a significantly greater risk of transient neurological symptoms following cervical manipulation (pooled relative risk = 1.96, 95% confidence interval 1.09–3.54, *p* < 0.05), but no greater risk of increased neck pain. The strength of this evidence was however reduced by limitations in the included studies [249].

The association of benign adverse events with patient outcomes is also not fully understood. In patients with neck pain, one study reported poorer pain and disability outcomes and also lower patient satisfaction in those patients who reported benign adverse events following cervical manipulation [243]. However, a further study found that while neck pain outcomes were also poorer in the short term for patients who experienced adverse events, at 3 months there was no association between worse outcomes and adverse events [227].

#### Box 2. Predicting benign adverse events and patient outcome following manual interventions to the spine: Summary of implications for clinical practice.

 • Patients presenting with moderate to high levels of neck disability may have an approximately three times greater likelihood of experiencing transient neurological symptoms (dizziness, nausea, visual disturbance, tinnitus, extremity weakness or confusion) following manual cervical treatment compared to patients with mild levels of neck disability;

 • It is not clear whether particular manual interventions have a greater risk for benign adverse events. Cervical manipulation may carry a greater risk compared with cervical mobilisation or thoracic manipulation in patients with neck pain. Non-specific effects or natural progression may also contribute to reporting of benign adverse events;

 • In neck pain patients, benign adverse events may result in poorer short-term outcomes, but do not seem to influence longer-term outcome.

#### Serious adverse events

RCTs and prospective cohort studies investigating the risks associated with manual spinal therapy for back and/or neck pain have not detected any serious adverse events following treatment [204, 207, 215, 222, 228, 231, 232, 234, 242, 243, 246, 247]. This suggests that serious adverse events are rare. Consequently, accurate calculations of risk rates are problematic, but failure to detect serious events does not confirm zero risk. Their design (sample size) renders RCTs and many cohort studies unlikely to capture very rarely occurring adverse events. Furthermore, in RCTs, strict inclusion/exclusion criteria and standardisation of treatments means that participants may not reflect the heterogeneity of real patient populations (e.g. their comorbidities) or of the treatments that they receive [286, 287].

Reported estimates of the incidence of serious adverse events vary, with estimates ranging from 1 per 2 million manipulations [276] up to 13 per 10,000 patients. Variation may be due to different statistical methods of estimation, estimates being based on calculations from different sized samples, evaluation of different types of patient, intervention or adverse event, and the fact that incidence may be calculated for serious adverse events following one manipulation, one visit, or per patient over several visits. A systematic review that pooled and analysed existing data utilised a method for estimating the risk of a major adverse event, expressing the upper 95% confidence interval. Risk estimated from cohort studies was approximately 0.01% (1 per 10,000) patients or 0.007% (7 per 100,000) treatments, when the estimate was based on zero cases from 22,833 patients receiving 42,451 treatments. Risk estimated from RCTs was approximately 0.13% (13 per 10,000) patients, based on zero cases from 2301 patients. While estimates indicate the relative rarity of serious adverse events, there are nevertheless a number of retrospective surveys [23, 142, 212, 239], case reports [19–184] and systematic reviews of case reports [266, 272, 278] describing serious complications following manual interventions to the spine.

The more frequently reported serious adverse events, attributed either to spinal manipulation or to chiropractor visits, include spinal cord injury following cervical, thoracic or lumbar manipulation (myelopathy, quadriplegia, paraplegia or Brown-Sequard syndrome) [20, 27, 29, 35, 38, 46, 47, 67, 73, 77, 90, 102, 105, 106, 108, 118, 125, 126, 139, 146, 154, 162, 167, 169, 171, 177, 183, 184, 190, 194, 197], cauda equina syndrome [19, 27, 29, 43, 58, 64, 109, 125, 143, 195, 203], dural tears (resulting in intracranial hypotension) [49, 50, 61, 68, 70, 95, 111, 119, 140, 163, 165, 186, 189, 193], epidural haemotomas [99, 101, 104, 146, 153, 159, 166, 168, 173, 184, 197, 288], pneumothorax or haematothorax [273], exacerbation of lumbar disk herniations [19, 23, 27, 29, 58, 74, 92, 109, 139, 143, 200, 203, 270] and, in relation to the cervical spine, cervical artery dissections [23–26, 28, 30, 32–34, 36, 37, 40, 42, 44–48, 51, 53, 54, 56, 57, 59–63, 66, 72, 76, 79–82, 84–86, 88, 89, 91–94, 96, 97, 100, 107, 108, 112, 113, 115–117, 120, 122–124, 127, 129, 130, 132–136, 138, 141, 142, 144, 148, 149, 152, 155, 161, 162, 170, 172, 174, 176, 178–182, 187, 188, 192, 196, 199, 201, 202] and exacerbation of cervical disk herniations [39, 55, 73, 92, 121, 126, 139, 169, 177, 191]. Serious neurological consequences of spinal nerve root injury are also reported, including diaphragmatic paralysis resulting from C3–5 (cervical spinal nerves) injury [69, 83, 114, 128, 131, 145, 151, 158]. Reporting of serious adverse events in the literature typically takes the form of either case reports or retrospective surveys. The principle limitation of what can be inferred from these is the difficulty of establishing causal relationships between the intervention and the adverse event. While a causative association cannot be proven, it also cannot be discounted. Further limitations include potentially incomplete or inaccurate reporting of the patient presentation prior to receiving care (i.e. whether they had pre-existing risk factors or indicators of a pathological process already underway) and scant details of the care provider or the intervention received [278, 280]. These limitations may result from the fact that the adverse event is typically reported by the medical specialist who has subsequently managed the patient and not by the manual therapist who delivered the intervention.

The best study design for evaluating the association of rare adverse responses to interventions is the case-control study [289]. With this design, a group of patients that has the condition being investigated (the ‘case’ group) is identified. A comparison group that does not have the condition but that is otherwise as similar as possible (the ‘control group’), is also selected. Analysis measures the frequency of exposure to the intervention in both groups to determine whether more of the ‘cases’ received it compared with the ‘controls’. Of the serious adverse events that have been reported following spinal manipulation, only cervical artery dissection has been investigated in this way [208–211, 221, 226, 233], therefore there is no data to enable accurate estimates of the level of association for any of the other serious adverse events.

#### Box 3. The occurrence of serious adverse events following manual interventions to the spine: Summary of implications for clinical practice.

 • Serious adverse events appear to be rare and, as a result, estimates of the level of risk are problematic;

 • However, cases of serious adverse events, including serious spinal or neurological problems as well as strokes affecting arteries in the neck, have been reported;

 • Serious adverse events could result from pre-existing pathologies, therefore assessment for signs or symptoms of these is important;

 • Where a serious adverse event is thought to have occurred following manual spinal intervention, use of a patient safety incident reporting system enables dissemination of accurate case details.

#### Cervical arterial stroke

Eight articles reported six case-control or case-crossover studies and one re-analysis of existing data [208] that specifically examined the association of cervical arterial strokes with prior visits to a chiropractor [208–211, 214, 221, 226] or with spinal manipulative therapy [233]. There are some differences between these in the classification of cervical arterial stroke [208] or of the intervention (visits to a chiropractor has been reported to be a poor proxy measure, used in some studies, for whether cervical manipulation took place [221]). Nevertheless, most reported consistent findings whereby cervical artery dissection patients under 45 years of age were between 3 and 12 times more likely to have received chiropractic or spinal manipulative therapy than the control groups to which they were compared [208, 209, 211, 214, 226, 233]. A single, recent, case-control study did not report a significant association, but contained very few cervical artery dissection patients in the under 45 age group [221]. While case-control studies can demonstrate an association between an intervention and an adverse outcome, they cannot provide evidence that this association is causative. Three studies also examined the association between cervical artery stroke and visits to a primary care physician, reporting a similar [209] or greater [221] risk of vertebral artery stroke and a similar risk of carotid artery stroke [211] compared with chiropractor visits. It is proposed, therefore, that chiropractic care did not pose an excess risk of cervical artery stroke and that headache or neck pain from an ongoing cervical artery stroke may have caused people to seek care from either a chiropractor or medical physician [209, 211, 289]. Some recent evidence supporting this postulation exists, whereby carotid artery stroke was more strongly associated with both chiropractor and primary care physician visits when neck pain or headache were symptoms, compared to when they were not [211]. Whether or not there is a causative relationship between chiropractic and cervical artery stroke, the association that exists indicates the potential for patients who may have an ongoing stroke to present to practitioners who utilise spinal manipulation. It is proposed, therefore, that careful screening for signs or symptoms of cervical artery stroke is crucial in patients presenting with neck pain, headaches or prior to receiving cervical manipulation for any reason, particularly in the under 45 age group. In addition, it has also been recommended that patients should be screened, prior to cervical manipulation, for the presence of known risk factors for cervical artery dissection [290], since this may be present in the absence of any signs or symptoms.

#### Box 4. The association of cervical arterial strokes with manual interventions to the cervical spine: Summary of implications for clinical practice.

 • There is some association, in the under 45 years age group, between manual interventions and stroke affecting arteries in the neck, however this is similar to that for medical practitioner visits;

 • It is possible that the manual intervention did not cause the stroke, but that the stroke caused neck pain, for which the patient visited a practitioner;

 • It is essential that careful screening for known neck artery stroke risk factors, or signs or symptoms that there is a problem, is performed prior to manual treatment of the neck.

#### Serious adverse events in children

The few studies evaluating adverse events following chiropractic care in children indicate the occurrence of benign, mild-moderate adverse events (including soreness, headache, dizziness, vomiting and excessive crying [218, 224]. While the paucity of existing reports [291] suggests that serious adverse events are rare, systematic reviews of studies in infants and children [279] identified descriptions of serious neurological consequences (quadriplegia, paraplegia, impaired level of consciousness, brainstem/cerebellum signs and subarachnoid haemorrhage), fractures (atlanto-axial dislocation, legs, ribs), haematothorax and respiratory failure following treatment from a variety of manual therapy practitioners, including three deaths. In many of the serious cases, there was pre-existing pathology that included congenital disorders (amyoplasia, torticollis, osteogenesis imperfecta), disorders of the nervous system (spinal cord astrocytoma, history of cranial nerve signs) and head trauma. Careful screening for signs or symptoms of pre-existing pathology is, thus, essential before treating children. A recently updated systematic review and Delphi process to inform best practice care of children emphasises the need for a thorough case history and examination, and specifies red flags and other particular considerations of which clinicians should be aware when assessing and managing children [283].

#### Box 5. Adverse events in children following manual spinal interventions - Summary of implications for clinical practice.

 • Children may experience benign, mild-moderate adverse events following manual interventions to the spine (including soreness, headache, dizziness, vomiting and excessive crying);

 • Cases of serious adverse events in children that may have followed manual, spinal care, including serious neurological or skeletal consequences, have been reported;

 • It is possible in some cases that the child had pre-existing pathology. Conducting a thorough case history and examination is thus essential before treating children.

#### Serious adverse events in older patients

Few studies have evaluated adverse events following manual spinal care in elderly patients. A single RCT evaluating adverse events in elderly participants with chronic neck pain reported that musculoskeletal adverse events were common with both spinal manual treatment and exercise interventions [245]. Case reports of serious adverse events in elderly patients, including osteoporosis-related compression fractures, do exist [65, 257]. A retrospective survey of 6,669,603 patients, aged between 66 and 99 years, with a visit to either a chiropractor or a primary care physician for a neuromusculoskeletal condition, evaluated the risk of traumatic injury to the head, neck or trunk presenting within the following 7 days [235]. Overall, the risk following a chiropractic visit was lower than that following a visit to a primary care physician, however, the likelihood of injury following chiropractic care was increased among patients with a chronic coagulation defect, inflammatory spondylopathy, osteoporosis, aortic aneurysm and dissection, or long-term use of anticoagulant therapy. A recently updated systematic review and Delphi process to inform best practice care of elderly patients concluded that there was no evidence for increased risk of serious adverse events, compared with the adult population in general, but specifies red flags and other particular considerations of which clinicians should be aware in the assessment and management of elderly patients [282]. These specific risk factors should therefore be considered when evaluating elderly patients prior to manual interventions.

A further report carried out a similar comparison in 1,157,475 neck pain patients of the same age group for risk of cervical artery stroke following chiropractic or primary care physician visits, concluding that there was little difference [236]. This is in accordance with case-control evidence evaluating cervical artery stroke following spinal manipulative therapy which indicates an association only in younger patients (under the age of 45) [289].

#### Box 6. Adverse events in elderly patients following manual spinal interventions - Summary of implications for clinical practice.

 • There does not seem to be any greater risk of traumatic injury for elderly patients visiting a chiropractor compared with visiting a medical practitioner for neuro-musculoskeletal problems;

 • Some underlying conditions may increase risk. It is essential to screen carefully for any such potential risk factors before treating elderly patients.

## Discussion

### Implications for clinical practice and for the relevant professions

A sizeable body of literature, with primary aims of evaluating safety and risks of manual treatment to the spine, was identified and characterised. Summaries of reported findings that may have implications for clinical practice (e.g. obtaining informed consent, assessment of patients for risk factors or indicators of underlying pathology) were compiled. However, limitations inherent in the design of studies that evaluate adverse events makes it difficult to establish firm conclusions.

The existing literature has implications for manual therapists in terms of communicating the risk of adverse events to patients. Evidence from the chiropractic profession suggests that many clinicians do not adequately communicate the risks of serious adverse events to their patients [292, 293]. While they agree that disclosure of risks is a moral and ethical part of care, concerns about increasing patient anxiety and possible refusal of care prevent them from doing so, even though there is evidence that the refusal of care rate following risk disclosure is low [294]. Chiropractic patients were found to perceive informed consent as a process and can be educated about the risks associated with treatment while satisfying the legal requirements of informed consent [295] and there is evidence that patients benefit from effective, informed decision- making [2, 3]. An important area for future research is to investigate how risk information may best be communicated to patients prior to receiving manual spinal care.

In addition to the implications for clinical practice that are described above, the relevant professions should adopt accurate reporting of cases where adverse events have occurred to provide a clearer understanding of the relevant facts. One mechanism for reporting is the use of patient safety incident reporting systems where clinicians may anonymously describe the circumstances around adverse events, enabling direct dissemination of information to peers and analysis by system operators. The Chiropractic Patient Incident Reporting and Learning System (CPiRLS) provides such a tool, enabling chiropractors to share their collective experience of adverse events and the system operators to develop and publish safer practice measures based on reporting trends [www.cpirls.org] [296].

### Implications for future research

Benign adverse events following manual spinal treatments have been relatively well characterised among adult patients. Evidence has been rated as ‘high quality’, based on consistent findings of both RCTs and observational studies that transient benign adverse effects are common [271]. There are, however, gaps in the available literature relating to prediction of adverse events. While there are some indications for the role of baseline symptom characteristics in predicting adverse events in neck pain patients [244], this has not been investigated among patients with spinal pain in other regions. Some studies report that the type of manual spinal treatment applied may predict the occurrence of benign adverse events, however indirectness in comparisons between studies and inconsistency in findings [244, 246, 247, 249] limit understanding. Further well-designed RCTs could establish causality between different interventions and benign adverse events, but due to their lack of generalisability to real patient populations, should be considered alongside observational studies. There is limited literature available relating to the occurrence of benign adverse events in special patient populations. Among elderly patients, only one RCT has evaluated the occurrence of benign adverse events following cervical manipulation; treatments to other spinal regions or in patients with other presenting conditions have not been studied. Among children, only one retrospective study has evaluated the occurrence of benign adverse effects [224]. Further prospective studies are needed to enable the responses of children to spinal manual treatments to be better understood.

The incidence and causal relationships between manual spinal treatments and serious adverse events are very challenging to establish due to the inherent methodological limitations of studies published to date. While a range of case-reports, case-series and case-reviews suggest that serious, sometimes catastrophic medical conditions have arisen in patients who have received manual spinal treatments, their methodological limitations mean that causality or non-causality cannot be established. RCTs and cohort studies are unlikely to detect the occurrence of very rare adverse events. The best study design to capture associations between interventions and rare adverse events is the case-control study. These have begun to elucidate associations between manual spinal treatment and cervical arterial stroke (the most commonly reported putative serious adverse event) in younger adults [208–211, 214, 221, 226, 233], but this study design cannot test causality and there are still issues interpreting the reported associations relating to methods of classification of strokes included [208] and to whether cervical manipulation was performed during recorded visits [221]. Other, more commonly reported, serious adverse events include intervertebral disk herniations, cauda equina syndrome, spinal cord injuries, dural tears associated with intracranial hypotension and phrenic nerve paralysis. However, there have been no investigations of association of these with spinal manual treatment utilising case-control study design, thus this relationship is unknown. Such studies also offer the possibility of stratification by age or other characteristics of participants, further elucidating the occurrence of serious adverse events in different patient populations.

Several secondary studies have taken the approach of pooling data from primary studies [249, 271] including RCTs, and cohort studies of clinical effectiveness. This can provide useful information from larger data sets, but necessitates consistent and accurate classification and reporting of adverse events in primary studies which has been reported to be limited [249, 271].

While 42 systematic reviews and meta-analyses were identified, some did appraise the quality of individual studies although, in reporting of findings, greater emphasis was placed on this for clinical outcomes than for adverse event outcomes. However, very few [249, 251, 269, 271, 281] graded quality across the body of evidence, limiting confidence in reported findings of other reviews. Future systematic reviews should therefore carry out thorough and transparent grading of both risk of bias in individual studies and also quality across the evidence reviewed [297, 298].

### Study limitations

The methodological framework for scoping reviews proposed by Arksey and O’Malley was followed for this review [18]. Within this, the principal limitation was that screening of records, selection for inclusion in the review and extraction of relevant data was performed by a single reviewer (for reasons of feasibility). A duplicate process would have increased confidence that studies were correctly included or excluded and that data were extracted accurately. The likelihood of incorrect study selection was reduced by adherence to detailed inclusion and exclusion criteria. However, some uncertainties were encountered in relation to assignation of reviews as systematic or non-systematic. This was due to the fact that some were not described as systematic reviews, yet did describe systematic approaches to some aspects of their methodology. Where this occurred, a conservative approach was taken of including all reviews that described, as a minimum, a systematic search strategy. This measure reduced the likelihood that valid studies failed to be included and the risk of omitting relevant information from the synthesis of findings.

A further limitation was that the quality of included studies, or of the body of evidence, was not appraised, although this is not considered essential to scoping reviews [14, 18]. The aim of this review was to characterise a broad and heterogenous body of literature relating to adverse events, whereas evidence quality appraisal usually addresses narrowly specified research questions [297]. Limitations in the evidence are described in the context of inherent weaknesses of study designs and gaps in the literature. However, it should be recognised that gaps in the evidence-base due to poor methodological quality within included studies are not identified.

The Arksey and O’Malley methodological framework for scoping studies [18] includes an optional final stage of a consultation exercise. This was not included here, but could have contributed to strengthening the focus on clinical implications, areas of uncertainty for clinicians and implementation of recommendations in practice.

## Conclusions

Benign adverse events are common following manual treatment to the spine [204, 207, 215, 222–225, 227, 228, 230–232, 234, 237, 242–244, 249, 250, 253, 256, 260, 262, 263, 276, 282]. These are usually mild and transient. Serious adverse events appear to be rare and are usually documented as case-reports, case series or retrospective surveys, making it difficult to quantify their occurrence or to establish causality. Nevertheless, there are reports of serious adverse events that may have followed manual treatments to the spine in both children and adults [19–184]. A greater body of evidence, in the form of case-control studies [208–211, 214, 221, 226, 233], indicates an association between chiropractic visits or spinal manipulation and vertebral artery stroke in younger adults, but also suggests that this may not be a causal relationship. There are substantial gaps in the literature regarding the association between manual spinal care and all other reported serious adverse events. It seems possible that pre-existing pathology may raise the risk of some of these events occurring, therefore detailed screening for known risk factors is essential prior to applying any manual spinal treatment to a patient of any age [282, 283, 290].

The existing literature has implications for manual therapists in terms of communicating the risk of adverse events to patients, and an important area for future research is to investigate how risk information may best be communicated to patients prior to receiving manual spinal care.

Clinicians can also help to elucidate uncertainties that arise around serious adverse events due to inaccurate case-reporting by disseminating their own case details first-hand. The use of patient safety incident reporting systems, such as CPiRLS [296], provide an anonymous way to share information where adverse events have occurred and to learn from these, and should be utilised routinely to enhance patient safety.

## Additional files

Additional file 1: Appendix 1. Example search strategy (MEDLINE and EMBASE). (PDF 318 kb)

Additional file 2: **Appendix 2.** Excluded records. (PDF 586 kb)

## Abbreviations

CPiRLS: Chiropractic patient incident reporting and learning system
RCT(s): Randomised controlled trial(s)
