# Supplementary material for: What are the risks of manual treatment of the spine? A scoping review for clinicians
Source: Chiropr Man Therap. 2017 Dec 7;25:37. doi: 10.1186/s12998-017-0168-5 (PMC5719861; doi:10.1186/s12998-017-0168-5)
Supplement: Supplementary file 1 — Appendix 1. Example search strategy (MEDLINE and EMBASE). (PDF 318 kb) [file 12998_2017_168_MOESM1_ESM.pdf]

APPENDIX 1 – Example search strategy (MEDLINE and EMBASE)

S1
(emb.exact("osteopathic care" OR "osteopathic manipulation" OR "osteopathic manipulative therapy" OR "osteopathic manipulative treatment" OR "osteopathic manual therapy" OR "osteopathic manual treatment" OR "osteopathic medicine" OR "osteopathic physician" OR "osteopathic spinal manipulation," OR "osteopathy" OR "osteopathy cranial" OR "chiropractic" OR "chiropractic joint manipulation therapy" OR "chiropractic manipulation" OR "chiropractic patient" OR "chiropractic medicine" OR "chiropractic physician" OR "chiropractic practitioner" OR "chiropractic spinal manipulation" OR "chiropractic spinal manipulative therapy" OR "chiropractor" OR "spinal manipulation" OR "spinal manipulation therapy" OR "spinal manipulative technique" OR "spinal manipulative therapy" OR "spinal manipulative treatment" OR "spinal manual technique" OR "spinal manual therapy" OR "naprapathy") OR mesh.exact("Chiropractic" OR "Manipulation, Chiropractic" OR "Manipulation, Osteopathic" OR "Manipulation, Spinal") OR TI,AB(chiropract[*2] OR osteopath[*2] OR naprapath[*2] OR "spinal manual therapy" OR "spinal manipulat[*3]" OR "vertebral manipulat[*3]" OR "spinal adjustment[*1]" OR "vertebral adjustment[*1]")) NOT "diabetic osteopathy"

S2
TI,AB("adverse event[*1]" OR "adverse effect[*1]" OR harm[*1] OR risk[*1] OR safe[*2] OR danger[*3] OR death[*1]) OR mesh.exact("adverse effects")

S3
TI,AB(stroke[*1] OR "vertebral arter[*3]" OR "carotid arter[*3]" OR "intra cranial hypotension" OR "dural tear[*1]" OR paralysis OR "brown sequard" OR "cauda equina")

S4
S2 OR S3

S5
(S1 AND S4)
