# Supplementary material for: What are the risks of manual treatment of the spine? A scoping review for clinicians
Source: Chiropr Man Therap. 2017 Dec 7;25:37. doi: 10.1186/s12998-017-0168-5 (PMC5719861; doi:10.1186/s12998-017-0168-5)
Supplement: Supplementary file 2 — Appendix 2. Excluded records. (PDF 586 kb) [file 12998_2017_168_MOESM2_ESM.pdf]

APPENDIX 2 - Excluded records

EXCLUDED FOLLOWING ABSTRACT SCREENING

Records not in English language

1. Tomé, F., A. Barriga, and L. Espejo, *Multiple disc herniation after chiropractic manipulation.* Revista de medicina de la Universidad de Navarra, 2004. **48**(3): p. 39-41.
2. An, G.-H., et al., *Effectiveness and safety of spinal manipulation for low back pain or neck pain: An overview of systematic reviews.* Chinese Journal of Evidence-Based Medicine, 2015. **15**(9): p. 1010-1017.
3. Brandt, T., E. Orberk, and C. Grond-Ginbach, *Clinical treatment and therapy for dissected cervicocerebral artery.* Nervenarzt, 2006. **77**(SUPPL. 1): p. S17-S30.
4. Tomé, F., A. Barriga, and L. Espejo, *Multiple disc herniation after chiropractic manipulation.* Archivos de Medicina del Deporte, 2005. **22**(107): p. 243-246.
5. Laniado-Laborín, R. and N. Cabrales-Vargas, *Diaphragmatic Paralysis Secondary to Chiropractic Manipulation of the Cervical Spine.* Revista del Instituto Nacional de Enfermedades Respiratorias, 2003. **16**(4): p. 254-256.
6. Lecocq, J. and P. Vautravers, *Complications of spinal manipulations.* Annales de Readaptation et de Medecine Physique, 1995. **38**(2): p. 87-94.
7. Schmitt, H.P., *Risks and complications of spinal manipulation: A neuropathological view.* Nervenarzt, 1988. **59**(1): p. 32-35.
8. Ernst, E., *Stroke after spinal manipulations? Commentary.* MMW-Fortschritte der Medizin, 2012. **154**(22): p. 33.
9. Ernst, E., *Risk of pediatric spinal manipulation.* MMW-Fortschritte der Medizin, 2007. **149**(14): p. 25.
10. Ernst, E., *How risky is cervical spine manipulation?* MMW-Fortschritte der Medizin, 2006. **148**(23): p. 26.
11. Lemmens, B., et al., *Phrenic nerve paralysis after cervical spine manipulation.* Presse médicale (Paris, France : 1983), 1992. **21**(35): p. 1685-1686.
12. Undabeitia, J., N. Samprón, and E. Úrculo, *Cauda equina syndrome after chiropractic treatment.* Neurocirugia, 2016. **27**(3): p. 151-153.
13. Palermo, M., et al., *Traumatic dissection of the extracranial right vertebral artery: Case report.* Rivista di Neurobiologia, 1997. **43**(5): p. 481-485.
14. Tinel, D., et al., *Vertebrobasilar ischemia after cervical spine manipulation: a case report.* Annales de readaptation et de medecine physique : revue scientifique de la Societe francaise de reeducation fonctionnelle de readaptation et de medecine physique, 2008. **51**(5): p. 403-14.
15. Menéndez-González, M., et al., *Wallenberg's syndrome secondary to dissection of the vertebral artery caused by chiropractic manipulation.* Revista de neurologia, 2003. **37**(9): p. 837-839.
16. Leboeuf-Yde, C., *How really dangerous is spinal manipulation?* Läkartidningen, 2000. **97**(4): p. 356, 359-361.
17. Rydell, N. and L. Räf, *Spinal manipulation--treatment associated with a high risk of complications.* Läkartidningen, 1999. **96**(34): p. 3536-3540.
18. *Adverse effects of alternative treatment.* Nordisk medicin, 1989. **104**(4): p. 123.
19. Chen, T.W. and S.T. Chen, *Brainstem stroke induced by chiropractic neck manipulation--a case report.* Zhonghua yi xue za zhi = Chinese medical journal; Free China ed, 1987. **40**(6): p. 557-562.

20. Schmitt, H.P., *Manual therapy in the region of the cervical spine. Manual therapy of the cervical spine and its dangers: ruptures and occlusions of the vertebral artery.* ZFA. Zeitschrift für Allgemeinmedizin, 1978. **54**(8): p. 467-474.
21. Tazelaar, G.H.P. and C.C. Tijssen, *Intracranial hypotension syndrome following manipulation of the cervical spine.* Nederlands Tijdschrift voor Geneeskunde, 2014. **158**(9).
22. Çitişli, V., et al., *Quadriplegia following chiropractic manipulation.* Journal of Neurological Sciences, 2012. **29**(3): p. 631-634.
23. Karl, I. and G. Egidi, *Chirotherapy - Conflicts between internal and external evidence.* Zeitschrift fur Allgemeinmedizin, 2012. **88**(7-8): p. 322-327.
24. Ritz-Timme, S., *Clarification of risks in chiropractic measures - Dissection of the arteria.* Rechtsmedizin, 2009. **19**(1): p. 63-69.
25. Kuituxiard, K., H.Z. Flach, and F. Van Kooten, *Bilateral vertebral artery dissection during chiropractic treatment.* Nederlands Tijdschrift voor Geneeskunde, 2008. **152**(45): p. 2464-2469.
26. Dreher, R., et al., *Insufficiency fractures in rheumatology. Case report and overview.* Zeitschrift fur Rheumatologie, 2006. **65**(5): p. 417-423.
27. Saxler, G., et al., *Spinal manipulative therapy and cervical artery dissections.* HNO, 2005. **53**(6): p. 563-567.
28. Izquierdo-Casas, J., et al., *Locked-in syndrome due to a vertebral dissection and therapeutic options with intraarterial fibrinolysis in acute phase.* Revista de Neurologia, 2004. **38**(12): p. 1139-1141.
29. Oehler, J., et al., *Bilateral vertebral artery dissection after chiropractic treatment.* Orthopade, 2003. **32**(10): p. 911-913.
30. Dupeyron, A., et al., *Complications following vertebral manipulation - A survey of a French region physicians.* Annales de Readaptation et de Medecine Physique, 2003. **46**(1): p. 33-40.
31. Yokota, J.-I., et al., *The medial medullary infarction (Dejerine syndrome) following chiropractic neck manipulation.* Brain and Nerve, 2003. **55**(2): p. 121-125.
32. Urakawa, M., et al., *Vertebral artery occlusion following neck trauma: Report of two cases.* Brain and Nerve, 2003. **55**(2): p. 141-145.
33. Gamer, D., et al., *Horner's syndrome in dissection of the carotid artery after chiropractic manipulation.* Klinische Monatsblatter fur Augenheilkunde, 2002. **219**(9): p. 673-676.
34. Cagnie, B., E. Vinck, and D. Cambier, *Side and adverse effects of spinal manipulation.* Tijdschrift voor Geneeskunde, 2002. **58**(20): p. 1317-1323.
35. Kraft, C.N., et al., *Non-cerebrovascular complications accompanying chiropractic manipulation of the cervical spine.* Zeitschrift fur Orthopadie und Ihre Grenzgebiete, 2001. **139**(1): p. 8-11.
36. Leweke, F., et al., *Bilateral vertebral dissection following chiropractic manipulation of the neck.* Aktuelle Neurologie, 1999. **26**(1): p. 35-39.
37. Bayer, K., *Vertebral artery dissection and chirotherapy.* Manuelle Medizin, 1998. **36**(5): p. 241-245.
38. Schilgen, M. and T. Graf-Baumann, *Vertebral artery injury and chiropractic manipulation of the cervical vertebrae spine: Practical consequences.* Manuelle Medizin, 1997. **35**(5): p. 249-253.
39. Ringelstein, E.B., *Dissections of the vertebral artery by chiropractic manipulation: An underestimated risk.* Manuelle Medizin, 1997. **35**(5): p. 240-245.
40. Alimi, Y., et al., *Dissecting aneurysm of vertebral arteries after chiropractic cervical manipulation. About two cases.* Journal des Maladies Vasculaires, 1996. **21**(5): p. 320-323.
41. Liepert, J., O. Rommel, and K. Witscher, *Electrophysiological findings in an iatrogenic case of Wallenberg's syndrome.* EEG-EMG Zeitschrift fur Elektroenzephalographie Elektromyographie und Verwandte Gebiete, 1995. **26**(4): p. 239-243.

42. Braus, D.F. and R. Mainka, *Stroke following chiropractic manipulation: A rational strategy for diagnosis.* Manuelle Medizin, 1993. **31**(4): p. 92-96.
43. Dupont, C., et al., *Two cases of locked-in syndrome after chiropractic cervical manipulation.* Annales de Readaptation et de Medecine Physique, 1992. **35**(2): p. 101-104.
44. Braune, H.J., M.H.J. Munk, and G. Huffmann, *Cerebral infarct in the area supplied by the medial cerebral artery after chiropractic manipulation of the neck.* Deutsche Medizinische Wochenschrift, 1991. **116**(27): p. 1047-1050.
45. Krieger, D., M. Leibold, and H. Bruckmann, *Dissections of the vertebral artery after cervical chiropractic manipulations.* Deutsche Medizinische Wochenschrift, 1990. **115**(15): p. 580-583.
46. Dahl, A., P. Bjark, and I.M. Anke, *Cerebrovascular complication following manipulation of the neck.* Tidsskrift for den Norske Laegeforening, 1982. **102**(3): p. 155-157+190.
47. Schmitt, H.P., *Ruptures and thromboses of the arteria vertebralis after closed mechanical injuries.* Schweizer Archiv fur Neurologie, Neurochirurgie und Psychiatrie, 1976. **119**(2): p. 363-379.
48. Schmitt, H.P. and L. Tamaska, *Dissecting rupture of vertebral artery with fatal thrombosis of vertebral and basilar arteries.* Zeitschrift fur Rechtsmedizin, 1973. **73**(4): p. 301-308.
49. Tachibana, S., K. Tashiro, and T. Kashiwaba, *A case of Wallenberg's syndrome after neck injury (Japanese).* Brain and Nerve, 1974. **26**(5): p. 603-607.
50. Wolff, H.D., *Cervical disc protrusion following chiropractic' therapy.* Man.Med, 1972. **10**(6): p. 124-127.
51. Oger, J., J. Brumagne, and J. Margaux, *Accidents in vertebral manipulation.* J. Belg. Med. Phys. Rhum., 1964. **19**(2): p. 56-78.
52. Oger, J., *Risks and accidents in vertebral manipulation.* Revue du rhumatisme, 1966. **33**(9): p. 493-504.
53. Grossiord, A., *Neurological accidents arising from cervical manipulation.* Annales de Medecine Physique, 1966. **9**(3): p. 283-298.

Excluded letters

54. *Osteopathic manipulation resulting in damage to the spinal cord.* British medical journal (Clinical research ed.), 1985. **291**(6510): p. 1720-1.
55. Bolton, J. and H. Thiel, *Adverse effects of spinal manipulation (6).* Journal of the Royal Society of Medicine, 2007. **100**(10): p. 446.
56. Brownhill, K., *Comments on "Spinal manipulation in patients with disc herniation: A critical review of risk and benefit".* International Journal of Osteopathic Medicine, 2007. **10**(1): p. 29.
57. Côté, P., et al., *Spinal manipulative therapy is an independent risk factor for vertebral artery dissection 1 (multiple letters).* Neurology, 2003. **61**(9): p. 1314-1315.
58. Cramer, G.D. and D.L. Smith, *Correctly identify practitioners and put adverse events of spinal manipulation into perspective.* Orthopedic Reviews, 2014. **6**(1): p. 20.
59. Di Duro, J.O., *Dural tear and intracranial hypotension in a chiropractic patient 8.* Journal of Neurology, Neurosurgery and Psychiatry, 2004. **75**(2): p. 346-347.
60. Dixon, P., *Adverse effects of spinal manipulation (1).* Journal of the Royal Society of Medicine, 2007. **100**(10): p. 444.
61. Dokos, C. and A. Tragiannidis, *Critique of review of deaths after chiropractic, 3.* International Journal of Clinical Practice, 2011. **65**(1): p. 103-104.
62. Ernst, E., *'First, do no harm' with complementary and alternative medicine.* Trends in Pharmacological Sciences, 2007. **28**(2): p. 48-50.

63. Ernst, E., *Adverse effects of spinal manipulation (8).* Journal of the Royal Society of Medicine, 2007. **100**(10): p. 447.
64. Grunnet-Nilsson, N., *Adverse effects of spinal manipulation (7).* Journal of the Royal Society of Medicine, 2007. **100**(10): p. 446.
65. Haldeman, S., et al., *Arterial dissections following cervical manipulation: the chiropractic experience.* CMAJ : Canadian Medical Association journal = journal de l'Association medicale canadienne, 2001. **165**(7): p. 905-6.
66. Haynes, M. and J. Sédat, *Stroke after chiropractic manipulation as a result of extracranial postero-inferior dissection 2 (multiple letters).* Journal of Manipulative and Physiological Therapeutics, 2003. **26**(8): p. 534-535.
67. Haynes, M., et al., *Internal forces sustained by the vertebral artery during spinal manipulative therapy 1 (multiple letters).* Journal of Manipulative and Physiological Therapeutics, 2004. **27**(1): p. 67-70.
68. Janati, A., *Carotid dissection.* Neurology, 1996. **47**(2): p. 610-1.
69. Johnson, I., *Adverse effects of spinal manipulation (3).* Journal of the Royal Society of Medicine, 2007. **100**(10): p. 444-445.
70. Lauretti, W.J., *Clarifying chiropractic manipulation risks.* CMAJ : Canadian Medical Association journal = journal de l'Association medicale canadienne, 2002. **166**(7): p. 886.
71. Lawrence, D.J., et al., *A risk/benefit analysis of spinal manipulation therapy for relief of lumbar or cervical pain 1.* Neurosurgery, 1994. **34**(3): p. 560-561.
72. Lewis, B.J., *Adverse effects of spinal manipulation (2).* Journal of the Royal Society of Medicine, 2007. **100**(10): p. 444.
73. Moore, A., *Adverse effects of spinal manipulation (4).* Journal of the Royal Society of Medicine, 2007. **100**(10): p. 445.
74. Myler, L., *A risk assessment of cervical manipulation vs. NSAIDs for the treatment of neck pain.* Journal of manipulative and physiological therapeutics, 1996. **19**(5): p. 357.
75. Nakamura, C.T., et al., *Vertebral artery dissection caused by chiropractic manipulation 2.* Journal of Vascular Surgery, 1991. **14**(1): p. 122-124.
76. Nilsson, N., W.J. Lauretti, and V. Dabbs, *A risk assessment of spinal manipulation vs. NSAIDs for the treatment of neck pain.* Journal of Manipulative and Physiological Therapeutics, 1996. **19**(3): p. 220-221.
77. Paterson, J.K., *Adverse effects of spinal manipulation (5).* Journal of the Royal Society of Medicine, 2007. **100**(10): p. 445-446.
78. Perle, S.M., S. French, and M. Haas, *Critique of review of deaths after chiropractic, 4.* International Journal of Clinical Practice, 2011. **65**(1): p. 104-105.
79. Perle, S.M. and J.M. Ventura, *RE: Vertebral Artery Dissection Presenting as Acute Cerebrovascular Accident.* Journal of Emergency Medicine, 2016. **50**(3): p. 506-507.
80. Persi, A., *Safety in chiropractic practice. Part II: Treatment to the upper neck and the rate of cerebrovascular incidents.* Journal of manipulative and physiological therapeutics, 1997. **20**(8): p. 566; author reply 566-567.
81. Poelsma, C. and D. Owen, *Critique of review of deaths after chiropractic, 2.* International Journal of Clinical Practice, 2011. **65**(1): p. 103.
82. Rosner, A., *Adverse events in the manipulation of pediatric patients: Flaws in a systematic review 19.* Pediatrics, 2007. **119**(6): p. 1261-1264.
83. Rosner, A.L., *Re: Chiropractic manipulation and stroke 3.* Stroke, 2001. **32**(9): p. 2207-2208.
84. Schneider, M., *Equivalent benefits/risks of cervical manipulation and mobilization.* Archives of Physical Medicine and Rehabilitation, 2011. **92**(2): p. 325.

85. Siegel, D. and T. Neiders, *Vertebral artery dissection and pontine infarct after chiropractic manipulation 8.* American Journal of Emergency Medicine, 2001. **19**(2): p. 171-172.
86. Snelling, N., *Reply to Comments on "Spinal manipulation in patients with disc herniation: A critical review of risk and benefit".* International Journal of Osteopathic Medicine, 2007. **10**(1): p. 30-31.
87. Turgut, M., *Ischemic stroke secondary to vertebral and carotid artery dissection following chiropractic manipulation of the cervical spine.* Neurosurgical Review, 2002. **25**(4): p. 267.
88. Wenban, A.B. and M. Bennett, *Critique of review of deaths after chiropractic, 1.* International Journal of Clinical Practice, 2011. **65**(1): p. 102.
89. Whedon, J.M., G.M. Bove, and M.A. Davis, *Critique of review of deaths after chiropractic, 5.* International Journal of Clinical Practice, 2011. **65**(1): p. 105.
90. Wolf, T.R. and S. Iyadurai, *Uncommon presentation of post chiropractic internal carotid artery dissection.* Neurosurgery, 2010. **67**(3): p. E878.

EXCLUDED FOLLOWING FULL TEXT APPRAISAL

Commentaries/editorials/opinion pieces

91. Cleland, J.A., *Spinal manipulation: Risks vs. benefits.* Southern Medical Journal, 2007. **100**(2): p. 132-133.
92. Ellrodt, A., *Assessing the risks of cervical manipulation for neck pain.* CMAJ : Canadian Medical Association journal = journal de l'Association medicale canadienne, 2002. **166**(9): p. 1134-1135.
93. Ernst, E., *Spinal manipulation: its safety is uncertain.* CMAJ : Canadian Medical Association journal = journal de l'Association medicale canadienne, 2002. **166**(1): p. 40-1.
94. Ernst, E., *Chiropractic spinal manipulation for back pain.* British journal of sports medicine, 2003. **37**(3): p. 195-6; discussion 196.
95. Ernst, E., *Spinal manipulation: Are the benefits worth the risks?* Expert Review of Neurotherapeutics, 2007. **7**(11): p. 1451-1452.
96. Ernst, E., *The safety of chiropractic cervical manipulation.* Focus on Alternative and Complementary Therapies, 2008. **13**(1): p. 41.
97. Haneline, M.T. and R. Cooperstein, *Internal carotid artery dissection with associated oculosympathetic palsy.* The Mount Sinai journal of medicine, New York, 2005. **72**(6): p. 421; author reply 422.
98. Haneline, M.T., A.C. Croft, and B.M. Frishberg, *Association of internal carotid artery dissection and chiropractic manipulation.* Neurologist, 2003. **9**(1): p. 35-44.
99. Haynes, M., *Risk of vertebrobasilar stroke and chiropractic care: results of a population based case control and case-crossover study.* Spine, 2011. **36**(1): p. 92; author reply 92.
100. Homola, S., *Neck manipulation, stroke and the precautionary principle.* Focus on Alternative and Complementary Therapies, 2014. **19**(4): p. 208-211.
101. Homola, S., *Pediatric Chiropractic Care: The Subluxation Question And Referral Risk.* Bioethics, 2016. **30**(2): p. 63-8.
102. Johnston, J., *Spinal manipulation by chiropractors is safe.* Canadian family physician Médecin de famille canadien, 1994. **40**: p. 434.
103. Jones, J., *Neurologists warn about link between chiropractic, stroke.* CMAJ : Canadian Medical Association journal = journal de l'Association medicale canadienne, 2002. **166**(6): p. 794.
104. Lawrence, D.J., *More data are necessary to understand the risk of adverse events following chiropractic manipulation.* Focus on Alternative and Complementary Therapies, 2009. **14**(4): p. 314-315.

105. Lawrence, D.J. and E. Ernst, *Spinal manipulation for neck pain - More good than harm?* Focus on Alternative and Complementary Therapies, 2004. **9**(2): p. 107-110.
106. Liberati, C., et al., *Spinal epidural hematoma as complication in chiropractic and acupuncture: Personal experience and literature review.* European Spine Journal, 2012. **21**(4): p. 800.
107. Maigne, J.-Y., *Cassidy JD, Boyle E, Côté P, et al. Risk of vertebrobasilar stroke and chiropractic care: results of a population-based case-control and case-crossover study. Spine 2008; 33(suppl 4):S176-S83.* Spine, 2008. **33**(25): p. 2838; author reply 2838-2839.
108. Moore, A., *Adverse effects of spinal manipulation.* Journal of the Royal Society of Medicine, 2007. **100**(10): p. 445; author reply 447.
109. Morrison, M.C., *Risks of manipulation.* Journal of the Royal Society of Medicine, 1993. **86**(3): p. 181.
110. Murphy, D.R., et al., *Does case misclassification threaten the validity of studies investigating the relationship between neck manipulation and vertebral artery dissection stroke? No.* Chiropractic and Manual Therapies, 2016. **24**(1).
111. Paulus, J.K. and D.E. Thaler, *Does case misclassification threaten the validity of studies investigating the relationship between neck manipulation and vertebral artery dissection stroke? Yes.* Chiropractic and Manual Therapies, 2016. **24**(1).
112. Plaugher, G., *Compression fractures in patients undergoing spinal manipulative therapy.* Journal of manipulative and physiological therapeutics, 1993. **16**(3): p. 193-5.
113. Rosner, A., *Adverse events in the manipulation of pediatric patients: flaws in a systematic review.* Pediatrics, 2007. **119**(6): p. 1261-4; author reply 1266-7.
114. Rosner, A.L., *Chiropractic: More good than harm or vice versa?* Journal of Manipulative and Physiological Therapeutics, 1999. **22**(4): p. 250-253.
115. Sangle, P.D., et al., *Internal carotid aneurysm presenting as hypoglossal and glossopharyngeal nerve palsy.* Clinical radiology, 2002. **57**(3): p. 233-4.
116. Vogel, S., *Adverse events and treatment reactions in osteopathy.* International Journal of Osteopathic Medicine, 2010. **13**(3): p. 83-84.
117. Vohra, S., *Children and chiropractic: What's the harm?* Focus on Alternative and Complementary Therapies, 2004. **9**(1): p. 12-14.
118. Wand, B.M., P.J. Heine, and N.E. O'Connell, *Should we abandon cervical spine manipulation for mechanical neck pain? Yes.* BMJ (Clinical research ed.), 2012. **344**: p. e3679.
119. Weintraub, M.I., *Spinal manipulative therapy is an independent risk factor for vertebral artery dissection.* Neurology, 2003. **61**(9): p. 1314; author reply 1314-1315.
120. Wilson, P.J., *Risks of manipulation.* Journal of the Royal Society of Medicine, 1993. **86**(3): p. 181.
121. Wilson, P.J., *Risks of manipulation.* Journal of the Royal Society of Medicine, 1994. **87**(12): p. 797.
122. Wright, G.T., *Assessing the risks of cervical manipulation for neck pain.* CMAJ : Canadian Medical Association journal = journal de l'Association medicale canadienne, 2002. **166**(9): p. 1134.

Non-systematic reviews

123. Vick, D.A., C. McKay, and C.R. Zengerle, *The safety of manipulative treatment: review of the literature from 1925 to 1993.* The Journal of the American Osteopathic Association, 1996. **96**(2): p. 113-5.
124. Tuchin, P., *Chiropractic and stroke: association or causation?* International journal of clinical practice, 2013. **67**(9): p. 825-33.

125. Rubinstein, S.M., *Adverse Events Following Chiropractic Care for Subjects With Neck or Low-Back Pain: Do The Benefits Outweigh the Risks?* Journal of Manipulative and Physiological Therapeutics, 2008. **31**(6): p. 461-464.
126. Paciaroni, M. and J. Bogousslavsky, *Cerebrovascular complications of neck manipulation.* European Neurology, 2009. **61**(2): p. 112-118.
127. Murphy, D.R., *Cervical manipulation and the myth of stroke.* Medicine and health, Rhode Island, 2012. **95**(6): p. 176-177.
128. Murphy, D.R., *Current understanding of the relationship between cervical manipulation and stroke: What does it mean for the chiropractic profession?* Chiropractic and Osteopathy, 2010. **18**.
129. Kerry, R., et al., *Manual therapy and cervical arterial dysfunction, directions for the future: A clinical perspective.* Journal of Manual and Manipulative Therapy, 2008. **16**(1): p. 39-48.
130. Humphreys, B.K., *Possible adverse events in children treated by manual therapy: A review.* Chiropractic and Osteopathy, 2010. **18**.
131. Homola, S., *Chiropractic, cervical spine manipulation, and stroke.* Scientific Review of Alternative Medicine, 2007. **11**: p. 19-22.
132. Gibbons, P. and P. Tehan, *HVLA thrust techniques: What are the risks?* International Journal of Osteopathic Medicine, 2006. **9**(1): p. 4-12.
133. Gibbons, P. and P. Tehan, *Spinal manipulation: Indications, risks and benefits.* Journal of Bodywork and Movement Therapies, 2001. **5**(2): p. 110-119.
134. Ernst, E., *Chiropractic: A Critical Evaluation.* Journal of Pain and Symptom Management, 2008. **35**(5): p. 544-562.
135. Ernst, E., *Chiropractic care: Attempting a risk-benefit analysis.* American Journal of Public Health, 2002. **92**(10): p. 1603-1604.
136. Ernst, E., *Complementary and alternative medicine in rheumatology.* Best Practice and Research: Clinical Rheumatology, 2000. **14**(4): p. 731-749.
137. Crawford, J.P., et al., *Vascular ischemia of the cervical spine: a review of relationship to therapeutic manipulation.* Journal of manipulative and physiological therapeutics, 1984. **7**(3): p. 149-155.
138. Biller, J., et al., *Cervical arterial dissections and association with cervical manipulative therapy: a statement for healthcare professionals from the american heart association/american stroke association.* Stroke, 2014. **45**(10): p. 3155-74.

Trial protocol

139. Walker, B.F., et al., *Outcomes of usual chiropractic, harm & efficacy, the ouch study: Study protocol for a randomized controlled trial.* Trials, 2011. **12**.

Conference abstract (not a case-report)

140. Khoo, S. and S. Dimmick, *Injury patterns post spinal manipulation.* Journal of Medical Imaging and Radiation Oncology, 2013. **57**: p. 147.

Did not report upon adverse events (biomechanical, haemodynamic or kinematic studies

141. Sran, M.M., et al., *Failure characteristics of the thoracic spine with a posteroanterior load: investigating the safety of spinal mobilization.* Spine, 2004. **29**(21): p. 2382-8.

142. Licht, P.B., et al., *Vertebral artery flow and cervical manipulation: An experimental study.* Journal of Manipulative and Physiological Therapeutics, 1999. **22**(7): p. 431-435.
143. Licht, P.B., et al., *Vertebral artery flow and spinal manipulation: A randomized, controlled and observer-blinded study.* Journal of Manipulative and Physiological Therapeutics, 1998. **21**(3): p. 141-144.
144. Erhardt, J.W., *Vertebral artery haemodynamics during atlantoaxial joint manipulation: A pilot study.* Journal of Manual and Manipulative Therapy, 2011. **19**(4): p. 241.
145. Cagnie, B., et al., *Changes in cerebellar blood flow after manipulation of the cervical spine using Technetium 99m-ethyl cysteinate dimer.* Journal of manipulative and physiological therapeutics, 2005. **28**(2): p. 103-7.
146. Cattrysse, E., et al., *Intended and non-intended kinematic effects of atlanto-axial rotational high-velocity, low-amplitude techniques.* Clinical biomechanics (Bristol, Avon), 2015. **30**(2): p. 149-52.
147. Buzzatti, L., et al., *Atlanto-axial facet displacement during rotational high-velocity low-amplitude thrust: An in vitro 3D kinematic analysis.* Manual therapy, 2015. **20**(6): p. 783-9.

Did not have adverse events as their primary aim

148. Touzé, E., et al., *Early asymptomatic recurrence of cervical artery dissection: three cases.* Neurology, 2003. **61**(4): p. 572-4.
149. Sran, M.M. and K.M. Khan, *Is spinal mobilization safe in severe secondary osteoporosis? - a case report.* Manual therapy, 2006. **11**(4): p. 344-51.
150. Sherrod, C., D. Johnson, and B. Chester, *Safety, tolerability and effectiveness of an ergonomic intervention with chiropractic care for knowledge workers with upper-extremity musculoskeletal disorders: a prospective case series.* Work (Reading, Mass.), 2014. **49**(4): p. 641-51.
151. Schneider, M., S. Weinstein, and G.P. Chimes, *Cervical manipulation for neck pain.* PM & R : the journal of injury, function, and rehabilitation, 2012. **4**(8): p. 606-12.
152. Saeed, A.B., et al., *Vertebral artery dissection: warning symptoms, clinical features and prognosis in 26 patients.* The Canadian journal of neurological sciences. Le journal canadien des sciences neurologiques, 2000. **27**(4): p. 292-6.
153. Rubinstein, S.M. and S. Haldeman, *Cervical manipulation to a patient with a history of traumatically induced dissection of the internal carotid artery: a case report and review of the literature on recurrent dissections.* Journal of manipulative and physiological therapeutics, 2001. **24**(8): p. 520-5.
154. Murphy, D.R., E.L. Hurwitz, and A.A. Gregory, *Manipulation in the presence of cervical spinal cord compression: a case series.* Journal of manipulative and physiological therapeutics, 2006. **29**(3): p. 236-44.
155. Menon, R.K. and J.W. Norris, *Cervical arterial dissection: Current concepts*. Annals of the New York Academy of Sciences. 2008: Blackwell Publishing Inc., 350 Main Street, MA 02148. 200-217.
156. Lucas, N.P. and R. Moran, *Clinical guidelines, adverse events and SQUID.* International Journal of Osteopathic Medicine, 2009. **12**(2): p. 47-48.
157. Lisi, A.J., E.J. Holmes, and C. Ammendolia, *High-velocity low-amplitude spinal manipulation for symptomatic lumbar disk disease: A systematic review of the literature.* Journal of Manipulative and Physiological Therapeutics, 2005. **28**(6): p. 429-442.
158. Lisi, A.J. and M.K. Bhardwaj, *Chiropractic high-velocity low-amplitude spinal manipulation in the treatment of a case of postsurgical chronic cauda equina syndrome.* Journal of manipulative and physiological therapeutics, 2004. **27**(9): p. 574-8.

159. Li, L., et al., *Cervical spine disease is a risk factor for persistent phrenic nerve paresis following interscalene block for shoulder surgery.* Regional Anesthesia and Pain Medicine, 2011. **36**(5).
160. Keramat, K.U. and A. Gaughran, *Safe physiotherapy interventions in large cervical disc herniations.* BMJ case reports, 2012. **2012**.
161. Jensen, T.W., *Vertebrobasilar ischemia and spinal manipulation.* Journal of manipulative and physiological therapeutics, 2003. **26**(7): p. 443-7.
162. Hing, W.A., D.A. Reid, and M. Monaghan, *Manipulation of the cervical spine.* Manual Therapy, 2003. **8**(1): p. 2-9.
163. Haneline, M. and J. Triano, *Cervical artery dissection. A comparison of highly dynamic mechanisms: Manipulation versus motor vehicle collision.* Journal of Manipulative and Physiological Therapeutics, 2005. **28**(1): p. 57-63.
164. Han, L., et al., *Short-term study on risk-benefit outcomes of two spinal manipulative therapies in the treatment of acute radiculopathy caused by lumbar disc herniation: study protocol for a randomized controlled trial.* Trials, 2015. **16**: p. 122.
165. Haldeman, S., et al., *Clinical perceptions of the risk of vertebral artery dissection after cervical manipulation: The effect of referral bias.* Spine Journal, 2002. **2**(5): p. 334-342.
166. Haas, M., M. Schneider, and D. Vavrek, *Illustrating risk difference and number needed to treat from a randomized controlled trial of spinal manipulation for cervicogenic headache.* Chiropractic and Osteopathy, 2010. **18**.
167. Grieve, G.P., *Contra-indications to spinal manipulation and allied treatments.* Physiotherapy, 1989. **75**(8): p. 445-453.
168. Gallerini, S., et al., *An unusual cause of cervicobrachial pain: vertebral artery dissection.* Neurological Sciences, 2017. **38**(6): p. 1111-1113.
169. French, S.D., B.F. Walker, and S.M. Perle, *Chiropractic care for children: Too much, too little or not enough?* Chiropractic and Osteopathy, 2010. **18**.
170. Evans, D.W., *Osteopathic principles: More harm than good?* International Journal of Osteopathic Medicine, 2013. **16**(1): p. 46-53.
171. Ernst, M.J., et al., *Extension and flexion in the upper cervical spine in neck pain patients.* Manual therapy, 2015. **20**(4): p. 547-52.
172. Ernst, E. and P. Posadzki, *Reporting of adverse effects in randomised clinical trials of chiropractic manipulations: A systematic review.* New Zealand Medical Journal, 2012. **125**(1353).
173. Ernst, E. and P.H. Canter, *A systematic review of systematic reviews of spinal manipulation.* Journal of the Royal Society of Medicine, 2006. **99**(4): p. 192-196.
174. Ernst, E., *Alternative medicine for pain: Trick or treatment.* European Journal of Pain Supplements, 2011. **5**(1): p. 1.
175. Ernst, E., *Complementary or alternative therapies for osteoarthritis.* Nature Clinical Practice Rheumatology, 2006. **2**(2): p. 74-80.
176. Downs, S.E., *Unilateral intermittent claudication of the left lower extremity.* Journal of manipulative and physiological therapeutics, 1988. **11**(4): p. 322-4.
177. Dougherty, P.E., et al., *Spinal manipulative therapy for elderly patients with chronic obstructive pulmonary disease: a case series.* J Manipulative Physiol Ther, 2011. **34**(6): p. 413-7.
178. Childs, J.D., T.W. Flynn, and J.M. Fritz, *A perspective for considering the risks and benefits of spinal manipulation in patients with low back pain.* Manual therapy, 2006. **11**(4): p. 316-20.
179. Alimi, Y.S., et al., *Blunt injury to the internal carotid artery at the base of the skull: six cases of venous graft restoration.* Journal of vascular surgery, 1996. **24**(2): p. 249-57.
180. Aktaruzzaman, M. and P. Zaman, *Allergy and asthma treatment by chiropractor: A hidden but substantial risk.* Annals of Allergy, Asthma and Immunology, 2010. **105**(5): p. A110.

Did not include spinal manual treatment

181. Yang, L., et al., *Efficacy and safety of chiropractic therapy in infantile anorexia: A systematic review.* European Journal of Integrative Medicine, 2016. **8**(2): p. 106-112.
182. Tissington Tatlow, W.F. and H.G. Bammer, *Syndrome of vertebral artery compression.* Neurology, 1957. **7**(5): p. 331-340.
183. Thaler, D.E., et al., *Dissection in the veterans' administration (DIVA): Re-examining spinal manipulation and cervical artery dissection.* Annals of Neurology, 2012. **72**: p. S13-S14.
184. Subramanian, S., et al., *Cauda equina syndrome without mechanical cauda compression: A report of 2 cases.* European Journal of Orthopaedic Surgery and Traumatology, 2008. **18**(4): p. 303-306.
185. Stence, N.V., et al., *Craniocervical arterial dissection in children: Diagnosis and treatment.* Current Treatment Options in Neurology, 2011. **13**(6): p. 636-648.
186. Sperry, K. and R. Pfalzgraf, *Inadvertent clavicular fractures caused by "chiropractic" manipulations in an infant: an unusual form of pseudoabuse.* Journal of forensic sciences, 1990. **35**(5): p. 1211-6.
187. Rose, D.Z. and M.R. Husain, *"Ostrich sign" indicates bilateral vertebral artery dissection.* Journal of stroke and cerebrovascular diseases : the official journal of National Stroke Association, 2012. **21**(8): p. 903.e1-2.
188. River, Y., et al., *Spontaneous vertebral artery dissection mimicking acute vertigo: Case report.* Annals of Otology, Rhinology and Laryngology, 1999. **108**(12): p. 1170-1173.
189. Rabkin, D.G., P. Benharash, and R.J. Shemin, *Vertebral artery dissection after iatrogenic cervical subcutaneous emphysema.* Journal of cardiac surgery, 2011. **26**(1): p. 54-6.
190. Probst, P., et al., *OMANT pilot trial - Randomised controlled pilot trial on feasibility, safety and effectiveness of osteopathic manipulative treatment following major abdominal surgery.* European Surgical Research, 2016. **57**(1-2): p. 80.
191. Probst, P., et al., *Randomised controlled pilot trial on feasibility, safety and effectiveness of osteopathic MANipulative treatment following major abdominal surgery (OMANT pilot trial).* International Journal of Osteopathic Medicine, 2016. **20**: p. 31-40.
192. Piovesan, E.J., et al., *Spontaneous vertebral artery dissection: A case report and review of the literature regarding clinical and radiological features.* Journal of the Neurological Sciences, 2015. **357**: p. e368.
193. Narancic Knez, N., M. Komso, and A. Mrdjen, *Carotid dissection and stroke-case report.* Cerebrovascular Diseases, 2015. **39**: p. 42.
194. Miyoshi, H., et al., *A case of coronary artery spasm caused by manipulation of the neck: heart rate variability analysis.* Journal of anesthesia, 2012. **26**(6): p. 905-9.
195. Leboeuf-Yde, C., L.R. Rasmussen, and N. Klougart, *The risk of over-reporting spinal manipulative therapy-induced injuries: a description of some cases that failed to burden the statistics.* Journal of manipulative and physiological therapeutics, 1996. **19**(8): p. 536-8.
196. Kier, A.L. and P.W. McCarthy, *Cerebrovascular accident without chiropractic manipulation: a case report.* Journal of manipulative and physiological therapeutics, 2006. **29**(4): p. 330-5.
197. Jao, T., et al., *Dissection of the posterior inferior cerebellar artery in a young adult with cerebellar infarct.* Acta neurologica Taiwanica, 2008. **17**(4): p. 243-7.
198. Ho, L., A.K.H. Lim, and F. Tanner, *Renal failure and acute interstitial nephritis associated with NSAIDs.* Journal of Pharmacy Practice and Research, 2005. **35**(3): p. 219-221.
199. Grammaticos, P., et al., *Cauda equina syndrome. An emergency, some unexpected severe symptoms and conservative treatment.* Hellenic Journal of Nuclear Medicine, 2016. **19**(3): p. 278-280.

200. Futch, D., et al., *Vertebral artery dissection in evolution found during chiropractic examination.* BMJ case reports, 2015. **2015**.
201. Fusco, M.R. and M.R. Harrigan, *Cerebrovascular dissections: A review. Part II: Blunt cerebrovascular injury.* Neurosurgery, 2011. **68**(2): p. 517-530.
202. Friedman, D.P. and A.E. Flanders, *Unusual dissection of the proximal vertebral artery: description of three cases.* AJNR. American journal of neuroradiology, 1992. **13**(1): p. 283-6.
203. Fedele, F.A., G. Ho Jr, and B.A. Dorman, *Pseudoaneurysm of the vertebral artery: A complication of rheumatoid cervical spine disease.* Arthritis and Rheumatism, 1986. **29**(1): p. 136-141.
204. Donahue, T.C. and J.C. Williams, *Greater saphenous vein and common femoral venous thrombosis in a recreational athlete: A case report.* Journal of Sports Chiropractic and Rehabilitation, 2000. **14**(1): p. 27-31.
205. Dhungana, S. and D.A. Adkins, *An unusual cause of vertebral artery dissection.* American Journal of Respiratory and Critical Care Medicine, 2015. **191**.
206. Derfus, B.A., et al., *Severe arthropathy and osteopathy following combined renal/pancreas transplantation.* Transplantation, 1992. **53**(3): p. 678-81.
207. Değirmenci, E., et al., *Wallenberg Syndrome following neck cracking: a case report.* European journal of physical and rehabilitation medicine, 2012. **48**(1): p. 167-8.
208. Davis, M.A. and J.A.M. Taylor, *A case of vertebral metastasis with pathologic c2 fracture.* Journal of manipulative and physiological therapeutics, 2007. **30**(6): p. 466-71.
209. Cristea, I. and C. Popa, *Spontaneous vertebral artery dissection with multiple supratentorial and infratentorial acute infarcts in the posterior circulation Case report.* Journal of medicine and life, 2016. **9**(3): p. 294-296.
210. Combs, S.B. and J.J. Triano, *Symptoms of neck artery compromise: case presentations of risk estimate for treatment.* Journal of manipulative and physiological therapeutics, 1997. **20**(4): p. 274-8.
211. Cagnie, B., et al., *A case of abnormal findings in the course of the vertebral artery associated with an ossified hyoid apparatus. A contraindication for manipulation of the cervical spine?* Journal of manipulative and physiological therapeutics, 2005. **28**(5): p. 346-51.
212. Burry, H.C. and V. Gravis, *Compensated back injury in New Zealand.* The New Zealand medical journal, 1988. **101**(852): p. 542-4.

Spinal manual therapy was not performed by a health professional

213. Quintana, J.G., et al., *Vertebral artery dissection and stroke following neck manipulation by Native American healer.* Neurology, 2002. **58**(9): p. 1434-5.
214. Mukherjee, S.T., *Cervical Manipulation Leading to Cerebellar Stroke in a Pilot.* Aerospace medicine and human performance, 2015. **86**(12): p. 1066-9.
215. Molumby, C. and J. Pasha, *Snap, crackle, stroke.* Journal of General Internal Medicine, 2015. **30**: p. S454.
216. Misra, U.K., J. Kalita, and D. Khandelwal, *Consequences of neck manipulation performed by a non-professional.* Spinal cord, 2001. **39**(2): p. 112-3.
217. Masneri, D.A., et al., *Trauma-induced pneumothorax after "bear-hug back crack" home remedy: attempted spinal manipulation by a layperson.* The Journal of the American Osteopathic Association, 2007. **107**(9): p. 411-3.
218. Johnson, D.W., G. Whiting, and M.P. Pender, *Cervical self-manipulation and stroke.* The Medical journal of Australia, 1993. **158**(4): p. 290.

Did not report upon patients

219. Dummett, J.F. and J. Langworthy, *Cauda Equina Syndrome (CES) and the communication of risk.* Manual Therapy, 2010. **15**(4): p. 408.
220. Rozmovits, L., S. Mior, and H. Boon, *Exploring approaches to patient safety: The case of spinal manipulation therapy.* BMC Complementary and Alternative Medicine, 2016. **16**(1).

Article was not possible to obtain

221. Bekavac, I., et al., *Chiropractic manipulation induced dissection and subsequent aneurysm formation of the internal carotid artery, or, if it ain't broke, don't fix it.* Explore (New York, N.Y.), 2006. **2**(2): p. 150-1.
222. Beran, R.G., A. Schaefer, and T. Sachinwalla, *Serious complications with neck manipulation and informed consent.* The Medical journal of Australia, 2000. **173**(4): p. 213-4.
